# Trends in incidence of proximal humerus fractures, surgical procedures and outcomes among elderly hospitalized patients with and without type 2 diabetes in Spain (2001–2013)

**DOI:** 10.1186/s12891-017-1892-7

**Published:** 2017-12-11

**Authors:** Maria Angeles Martinez-Huedo, Rodrigo Jiménez-García, Eduardo Mora-Zamorano, Valentín Hernández-Barrera, Manuel Villanueva-Martinez, Ana Lopez-de-Andres

**Affiliations:** 1 Preventive Medicine and Public Health, Unidad de Docencia, Hospital Universitario La Paz, Comunidad de Madrid, Madrid, Spain; 2 Preventive Medicine and Public Health Teaching and Research Unit, Health Sciences Faculty, Rey Juan Carlos University, Avda. de Atenas s/n, 28922 Alcorcón, Madrid, Spain; 3 Preventive Medicine and Public Health, Hospital Universitario de Torrejón, Comunidad de Madrid, Torrejón de Ardoz, Spain; 4 Avanfi, Instituto Avanzado en Medicina Deportiva, Traumatología, Podología y Fisioterapia, Comunidad de Madrid, Madrid, Spain

**Keywords:** Proximal humerus fracture, Type 2 diabetes, Surgical procedures, In-hospital mortality, Hospitalization outcomes

## Abstract

**Background:**

Several studies have reported that diabetic persons have an increased risk for fractures than non-diabetes patients. The association between proximal humerus fractures and type 2 diabetes (T2DM) is unclear and some studies point to insulin treatment, hypoglycaemic episodes consequently to inadequate control of diabetes or, more recently, to an alteration of trabecular bone. We examined trends in the incidence of proximal humerus fractures, surgical procedures and outcomes among hospitalized patients aged ≥65 years, with and without T2DM in Spain, 2001–2013.

**Methods:**

This retrospective, observational study was conducted using the Spanish National Hospital Discharge Database to select all hospital admissions with proximal humerus fracture. We calculated incidences overall and stratified by diabetes status, year and sex. We analyzed surgical procedures, comorbidities, length of stay, in-hospital complications and in-hospital mortality.

**Results:**

We identified 43,872 patients with proximal humerus fracture (18.3% had a T2DM diagnosis). Age-adjusted incidence rates elevated steadily over the study period for men and women with and without T2DM, independently of diabetes status, although we found a stable trend in the later years. Patients with T2DM had lower relative risk of proximal humeral fracture incidence: 0.87 (95%IC 0.82–0.93) for men and 0.97 (95%IC 0.95–1.00) for women. In-hospital complications were 4.0% of diabetic men vs. 2.6% in non-diabetic (*p* < 0.001) and 2.9% among T2DM women vs. 1.7% in those without (*p* < 0.05). The use of open reduction of fracture with internal fixation and arthroplasty is increasing overtime and closed reduction with internal fixation is decreasing. Presence of T2DM in women was associated with higher in-hospital mortality (OR 1.67; 95%CI 1.29–2.15). Comorbidities, in-hospital complications and older age were predictors of higher in-hospital mortality in both sexes.

**Conclusions:**

The incidence of proximal humerus fractures seems to be increasing in Spain. The incidence is lower among men with than without T2DM. T2DM is associated to higher in-hospital complications in both sexes. The use of open reduction of fracture with internal fixation and arthroplasty is increasing overtime beside diabetes status. Women with T2DM have higher in-hospital mortality than those without the disease.

**Electronic supplementary material:**

The online version of this article (10.1186/s12891-017-1892-7) contains supplementary material, which is available to authorized users.

## Background

It is well known that the incidence of diabetes mellitus is growing steadily all around the world, especially in older people and it is becoming a very important public health problem [1, 2]. Several studies have reported that diabetic persons have an increased risk for fractures than non-diabetes patients; however the underlying mechanisms for this difference in fracture risk are not clear [3].

The epidemiology and risk factors for proximal humerus fracture (PHF) have been previously described. [4–14].

PHF represent 10% of all fractures in people age 65 years or over [4–6]. These are the third most frequent fractures in patients ≥65 years, following wrist and femoral neck fractures, which are all considered pathologic fractures favored by osteoporosis [7, 8]. Some studies have found higher mortality in PHF than in other types of fractures [9, 10]. PHF show higher incidence among women [4–7, 9–13]. Risk factors associated with this type of fracture such as comorbidity, osteoporosis, older age and falls, are found in >75% of these fractures [7, 11]. Time trend analyses suggest that the incidence of PHF is becoming stable over the last years [8, 14].

The association between PHF and type 2 diabetes (T2DM) is unclear and some studies point to insulin treatment, hypoglycaemic episodes consequently to inadequate control of diabetes [1] or, more recently, to an alteration of trabecular bone. All these factors may predispose to this type of fracture despite normal or higher than normal bone mineral density [15–17]. However, a randomized trial did not find an association between inadequate control of diabetes and a higher risk of fractures [18].

Regarding the type of treatment used in PHF, several factors are important in determining the choices of treatment and include, among other, the pattern of the fracture (i.e. the number of segments), the displacement of these segments, patient characteristics (age, comorbidity, cognitive status, compliance), surgeon and implant availability, local traditions and guideline. [19]. However, in our country, like overseas, the indications are related to the nature and pattern of the fracture. 1 segment fractures are mostly non-operative treated. For 2 segments fractures treatment options include Closed Reductions of fracture with Internal Fixation (CRIF) or Open Reduction of fracture with Internal Fixation (ORIF). 3 and 4 segments fractures are usually treated with ORIF or Partial Shoulder Replacement (PSR).

Based on national hospital discharge data, we compared trends in the incidence of PHF among hospitalized elderly patients with and without T2DM in Spain from 2001 to 2013. We focused on trends in the type of surgical interventions and also described and analyzed patient comorbidities, in-hospital complications, and in-hospital outcomes such as: length of hospital stay (LOHS) and in-hospital mortality (IHM).

## Methods

We performed a retrospective, observational study using the Spanish National Hospital Database (SNHDD) [20]. We analyzed data collected between January 1, 2001 and December 31, 2013 for subject’s aged 65 and over.

The SNHDD database compiles all the public and private hospital data, hence covering more than 95% of hospital discharges [20].

The database includes patients’ variables (sex, date of birth), date of admittance, date of discharge, type of admission (elective or emergency admission), discharge destination (home, decease or other health/social institution), up to 14 discharge diagnosis, and up to 20 procedures performed during the admission [20].

If patients has not been hospitalized for over 24 h in a hospitalization ward it is not recorded in SNHDD [20]. Therefore, if patients are discharged from the emergency room, beside how long the patient has been there, or treated at out-patient clinic they are not included in the SNHDD and in this study.

We selected all patients hospitalized with a primary diagnosis of PHF (code ICD-9-CM: 812.xx). Only the first PHF per patient was countedelected for analysis.

We classified as patients with T2DM those who had in any diagnosis position the ICD-9-MC codes 250.×0 or 250.×2, patients with type 1 diabetes (T1DM) those who had in any diagnosis position the ICD-9-MC codes 250.×1 or 250.×3, and patients without diabetes those who did not have any of these codes in their discharge report. Patients with T1DM were excluded from the analysis.

Comorbidity was assessed by calculating the Charlson comorbidity index (CCI), excluding diabetes as a disease [21]. Patients were divided into three categories: CCI0 (patients with no previously recorded disease); CCI1, (patients with one disease category; and CCI ≥ 2 (patients with two or more disease categories).

The SNHDD does not include information on the characteristics of the fracture, such as one, two, three or four segments neither the degree of displacement. The only information is the procedures conducted according to the ICD-9-CM classification.

Procedures considered in the data analysis included: total shoulder replacement (TSR): ICD-9-CM code: 81.80 (replacement of right or left shoulder joint with autologous tissue substitute, synthetic substitute, non-autologous tissue substitute all using an open approach. This code includes all types of TSR, with or without tissue substitute, and excludes reverse total shoulder replacement). PSR ICD-9-CM code: 81.81. CRIF ICD-9-CM code: 79.11 (reposition of the humeral head with internal fixation device using percutaneous or endoscopic approach and the reposition of the humeral shaft with internal fixation device using percutaneous or endoscopic approach). ORIF ICD-9-CM codes: 79.31 (reposition of the humeral head or the humeral shaft with internal fixation device using an open approach).

TSR and PSR were combined in one variable “Arthroplasty”, as they were both prosthetic surgical procedures with similar results with regard to trends and in hospital outcomes.

If the patient did not have any of the previous was considered to have a conservative non-operative treatment (closed reduction and immobilization).

We analyzed adverse in-hospital events which included the development of one or more of any of the following in-hospital complications (IHC) recorded in any diagnosis positions: pneumonia (ICD-9-CM codes: 997.39, 486), sepsis (ICD-9-CM codes: 995.91, 995.92), acute renal failure (ICD-9-CM codes: 584, 584.5, 584.6, 584.7, 584.8, 584.9), surgical site infection (ICD-9-CM codes: 998.5, 998.51, 998.52, 998.53, 998.54, 998.55, 998.59, 998.50), iatrogenic pulmonary embolism and infarction (ICD-9-CM codes: 415.11) (It includes the diagnosis of an infarction of the lung after a iatrogenic pulmonary embolism. Possible reasons for a iatrogenic pulmonary embolism include i) Air embolism following infusion, transfusion and therapeutic injection, ii) embolism of cardiac prosthetic devices, implants and grafts, iii) embolism of vascular prosthetic devices, implants and grafts, iv) Complication of a vein or an artery following a procedure), deep venous thrombosis (ICD-9-CM codes: 453.4, 453.40, 453.41, 453.42), and urinary tract infection (ICD-9-CM codes: 599.0), as described previously [19].

The median LOHS and IHM were also calculated for each year studied.

### Statistical analysis

Age adjusted incidence rates of discharge after PHF were calculated for men and women with and without T2DM per 100,000 inhabitants using the methods and population estimates described previously [22–24]. We also calculated the Incidence Rate Ratios (IRR) of suffering a PHF for T2DM versus non-diabetes for men and women using Poisson Regression.

In order to identify the period in which trend changes in PHF rates occurred by sex and diabetes status we used log linear Joinpoint regression. We estimated the “annual percentage of change” in each of the periods delimited by the points of change [25]. Joinpoint Regression Program, version 4.0.4. was used for the analysis [26].

All study variables were described stratified according to T2DM status and sex. Categorical variables are expressed as proportions; continuous variables as means with their standard deviation or as medians with their IRQ (p25-p75).

The bivariate analysis of trends was performed by year using a χ^2^ linear trend test (proportions), ANOVA (means), and Kruskal-Wallis test (medians), as appropriate.

To compare proportions between those with and without T2DM we conducted logistic regression models adjusted by age and year. Mean and medians were compared with ANOVA and Kruskal-Wallis test respectively.

In the case of IHM, logistic regression analysis was performed, with mortality as a binary outcome using year of discharge, age, CCI, complications and type of treatment as independent variables for patients with and without T2DM, in men and women and for the population as a whole to assess the influence of T2DM on IHM. Statistical analysis was performed using Stata version 10.1 (Stata, College Station, Texas, USA). Statistical significance was set at *p* < 0.05 (2-tailed).

## Results

From 2001 through 2013 we selected 43,872 patients aged 65 years or older whose primary diagnosis on admission was PHF. The overall mean age was 76.17 years (SD 6.78 years) and 83% were women. Among these patients we identified 8049 suffering T2DM (18.3%), mean age of 76.21 years (SD 6.8 years) and 84.5% of them women, and 35,823 patients without diabetes, mean age was 75.98 years (SD 6.4 years) and 82.8% women.

Tables 1 and 2 show age adjusted incidence rates of PHF and clinical characteristics among men and women with and without T2DM. Incidence of PHF among men without T2DM increased significantly (*p* < 0.001) from 14.24 cases per 100.000 inhabitants in 2001 to 23.28 cases in 2013. Among men with T2DM we observed a significant (*p* < 0.001) increase from 11.47 cases per 100.000 inhabitants in 2001 to 20.30 cases in 2013.

**Table 1:** Age adjusted incidence rates and clinical characteristics of hospital discharges due to proximal humerus fracture among men with and without type 2 diabetes in Spain, 2001–2013

	2001	2002	2003	2004	2005	2006	2007	2008	2009	2010	2011	2012	2013	Total
NO T2DM														
Number	320	350	357	384	431	436	461	543	574	591	530	622	595	6194
Incidence^a^	14.24	15.51	14.92	16.33	17.45	17.30	18.3	22.03	22.08	22.73	20.01	23.37	23.28	19.01
Age, mean(SD)	74.8(6.3)	74.7(6.6)	75.2(6.8)	75.6(6.6)	75.1(6.5)	75.5(6.9)	74.9(6.6)	75.4(6.9)	76.0(6.9)	75.5(6.9)	75.5(7.1)	75.6(7.1)	75.2(7.1)	75.4(6.8)
CCI 0, n(%)^ab^	246(76.9)	271(77.4)	280(78.4)	282(73.4)	322(74.7)	328(75.2)	343(74.4)	391(72.0)	408(71.1)	418(70.7)	380(71.7)	429(69.0)	414(69.6)	4512(72.8)
CCI 1,n(%)^ab^	59(18.4)	63(18.0)	61(17.1)	85(22.1)	88(20.4)	82(18.8)	100(21.7)	123(22.7)	136(23.7)	133(22.5)	114(21.5)	144(23.2)	136(22.9)	1324(21.4)
CCI ≥2, n(%)^ab^	15(4,7)	16(4,6)	16(4,5)	17(4,4)	21(4,9)	26(6,0)	18(3,9)	29(5,3)	30(5,2)	40(6,8)	36(6,8)	49(7,9)	45(7,6)	358(5,8)
IHC, n(%)^b^	5(1.6)	6(1.7)	8(2.2)	11(2.9)	8(1.9)	12(2.8)	5(1.1)	26(4.8)	10(1.7)	14(2.4)	13(2.5)	25(4.0)	15(2.5)	158(2.6)
T2DM														
Number	42	42	61	59	77	81	90	105	115	132	147	148	148	1247
Incidence^a^	11.47	9.54	11.96	12.36	14.26	13.99	15.55	19.46	18.10	20.52	21.09	21.01	20.30	16.55
Age, mean(SD)	74.8(6.3)	73.9(5.7)	75.2(6.6)	76.2(6.7)	76.2(7.2)	74.5(6.6)	75.1(6.6)	74.8(6.3)	75.6(6.4)	76.4(6.8)	74.9(6.4)	75.7(6.3)	76.0(7.0)	75.4(6.8)
CCI 0, n(%)^a^	24(57.1)	31(73.8)	45(73.8)	38(64.4)	55(71.4)	51(63.0)	57(63.3)	73(69.5)	71(61.7)	71(53.8)	85(57.8)	92(62.2)	79(53.4)	772(61.9)
CCI 1, n(%)^a^	12(28.6)	9(21.4)	12(19.7)	17(28.8)	14(18.2)	23(28.4)	28(31.1)	25(23.8)	30(26.1)	41(31.1)	46(31.3)	41(27.7)	51(34.5)	349(28.0)
CCI ≥2, n(%)^a^	6(14.3)	2(4.8)	4(6.6)	4(6.8)	8(10.4)	7(8.6)	5(5.6)	7(6.7)	14(12.2)	20(15.2)	16(10.9)	15(10.1)	18(12.2)	126(10.1)
IHC, n(%)^a^	1(2.4)	1(2.4)	2(3.3)	1(1.7)	5(6.5)	1(1.2)	3(3.3)	2(1.9)	6(5.2)	5(3.8)	8(5.4)	6(4.1)	9(6.1)	50(4.0)

*N* Number of discharges, Incidence: per 100.000 inhabitants; CCI (Charlson Comorbidity Index): IHC: In-hospital complications ^a^ *P* < 0.05 Poisson regression for incidences trends, χ2 linear trend analysis for proportions and ANOVA for age. ^b^. *P* < 0.05 when comparing total values of study variables between men with and without T2DM. To compare proportions we conducted logistic regression models adjusted by age and year. Means were compared with ANOVA

**Table 2:** Age adjusted incidence rates and clinical characteristics of hospital discharges due to proximal humerus fracture among women with and without type 2 diabetes in Spain, 2001–2013

	2001	2002	2003	2004	2005	2006	2007	2008	2009	2010	2011	2012	2013	Total
NO T2DM														
Number	1605	1629	1731	1922	2098	2179	2270	2458	2617	2855	2685	2702	2878	29,629
Incidence^a^	54.27	55.19	54.15	60.32	62.94	63.26	65.9	74.08	73.57	80.42	74.40	75.22	79.33	67.30
Age, mean(SD)^a^	76.1(7.0)	76.2(6.8)	76.1(6.7)	76.3(6.7)	76.2(6.8)	76.5(6.7)	76.2(6.4)	76.6(6.7)	76.8(6.9)	76.6(6.8)	76.5(6.9)	76.2(6.9)	76.3(7.1)	76.4(6.8)
CCI 0, n(%)^ab^	1389(86.5)	1434(88.0)	1487(85.9)	1662(86.5)	1819(86.7)	1890(86.7)	1936(85.3)	2104(85.6)	2199(84.0)	2413(84.5)	2277(84.8)	2230(82.5)	2375(82.5)	25,215(85.1)
CCI 1, n(%)^ab^	199(12.4)	179(11.0)	214(12.4)	228(11.9)	237(11.3)	257(11.8)	294(13.0)	316(12.9)	366(14.0)	380(13.3)	337(12.6)	392(14.5)	419(14.6)	3818(12.9)
CCI ≥2, n(%)^ab^	17(1.1)	16(1.0)	30(1.7)	32(1.7)	42(2.0)	32(1.5)	40(1.8)	38(1.5)	52(2.0)	62(2.2)	71(2.6)	80(3.0)	84(2.9)	596(2.0)
IHC, n(%)^a, b^	15(0.9)	23(1.4)	31(1.8)	20(1.0)	25(1.2)	32(1.5)	35(1.5)	44(1.8)	45(1.7)	51(1.8)	54(2.0)	64(2.4)	58(2.0)	497(1.7)
T2DM														
Number	231	280	311	376	418	461	553	560	687	715	712	719	779	6802
Incidence^a^	32.54	40.72	43.92	52.43	57.97	64.20	77.02	78.10	85.72	89.13	80.10	80.94	87.42	67.23
Age, mean(SD)	75.7(6.5)	76.2(6.4)	75.8(6.6)	75.5(5.9)	75.5(6.1)	75.9(6.4)	76.5(6.6)	76.1(6.3)	76.0(6.2)	76.2(6.2)	76.2(6.4)	76.6(6.4)	75.9(6.6)	76.4(6.4)
CCI 0, n(%)^a^	187(81.0)	229(81.8)	246(79.1)	308(81.9)	342(81.8)	356(77.2)	419(75.8)	438(78,2)	533(77.6)	552(77.2)	537(75.4)	536(74.5)	596(76.5)	5279(77.6)
CCI 1, n(%)^a^	41(17.7)	44(15.7)	57(18.3)	63(16.8)	64(15.3)	90(19.5)	106(19.2)	93(16,6)	130(18.9)	138(19.3)	134(18.8)	143(19.9)	143(18.4)	1246(18.3)
CCI ≥2, n(%)^a^	3(1.3)	7(2.5)	8(2.6)	5(1.3)	12(2.9)	15(3.3)	28(5.1)	29(5,2)	24(3.5)	25(3.5)	41(5.8)	40(5.6)	40(5.1)	277(4.1)
IHC, n(%)^a^	5(2.2)	10(3.6)	7(2.3)	7(1.9)	10(2.4)	14(3.0)	17(3.1)	20(3,6)	21(3.1)	22(3.1)	17(2.4)	29(4.0)	21(2.7)	200(2.9)

*N* Number of discharges, Incidence: per 100.000 inhabitants, *CCI* (Charlson Comorbidity Index), *IHC* In-hospital complications ^a^ *P* < 0.05 Poisson regression for incidences, χ2 linear trend analysis for proportions and ANOVA for age ^b^. *P* < 0.05 when comparing total values of study variables between women with and without T2DM. To compare proportions we conducted logistic regression models adjusted by age and year. Means were compared with ANOVA

Incidence of PHF among women increased significantly among diabetic and non-diabetic along the study period. For non-diabetic women it increased from 54.27 cases per 100,000 inhabitants in 2001 to 79.23 cases in 2013 (*p* < 0.001). Similarly, among women suffering T2DM the incidence rose from 32.54 cases in 2001 to 87.42 cases in 2013 (*p* < 0.001).

The results of the Poisson regression yielded an IRR for diabetic patients versus non-diabetic patients for suffering a PHF of 0.87 (95%IC 0.82–0.93: *p* < 0.01) for men and 0.97 (95%IC 0.95–1.00; *p* < 0.01) for women. This means that over the entire period men with T2DM has a lower incidence of PHF than those without T2DM after adjusting by age. No significant differences were observed among women.

The Joinpoint analysis showed that age-adjusted PHF diagnosis in men without T2DM increased significantly throughout this period by 4.51% (95%IC 3.2–5.8;*p* < 0.05) each year (see Additional file 1: Figure S1). For men with T2DM it increased significantly by 8.38% (95%IC 7.1–9.6; *p* < 0.05) per year from 2001 to 2011 and from 2011 to 2013 showed a slightly non-significant declining trend −3.28%(95%IC -12.9-7.59) (see Additional file 2: Figure S2). Among women without T2DM the Joinpoint analysis showed that the incidence rate increased 4.94% (95%IC 4.2–5.7; *p* < 0.05) per year from 2001 to 2010 and from 2011 to 2013 kept a stable rate of −0.5% (95%IC -3.7-2.7) (see Additional file 3: Figure S3).

In women suffering T2DM, incidence of PHF diagnosis showed an abrupt increase from 2001 to 2007 of 16.32% (95%IC 11.7–21.2; *p* < 0.05) per year. However, from 2008 to 2013 there were no significant changes in the incidence (see Additional file 4: Figure S4).

Regarding CCI values, they were higher for diabetic than non-diabetic men (CCI ≥ 2, 10.1% vs 5.8% respectively) and also for IHC, where we observed complications in 4.0% of diabetic men vs. a 2.6% in non diabetic men (*p* < 0.001) (Table 1).

Again, we also observed higher values in CCI with two or more comorbidities and IHC among women with T2DM than women without diabetes (CCI ≥ 2, 4.1% vs. 2.0% and IHC 2.9% vs. 1.7%, *p* < 0.005) (Table 2) and higher IHC (2.9% vs. 1.7%; *p* < 0.05).

**Fig. 1:**
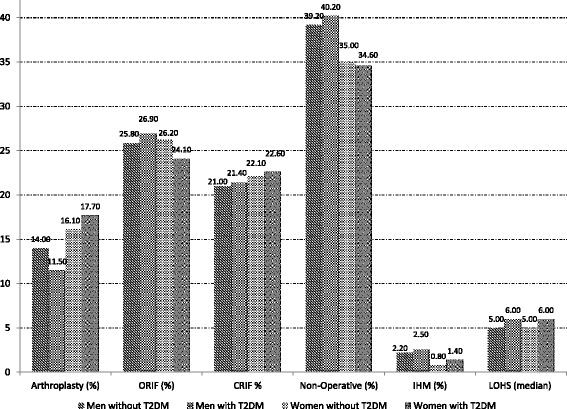
Procedures and outcomes of PHF among men and women with and without T2DM (2001–2013). Arthroplasty: Total or partial humerus replacement; ORIF: Open reduction of fracture with internal fixation; CRIF: Close reduction of fracture with internal fixation; IHM: In-hospital mortality; LOHS: Length of hospital stay

Figure 1 shows the procedures and in-hospital outcomes for men and women with a PHF, according to T2DM status over the entire study period. The detailed description year by year is shown in Additional file 5: Table S1.

We found that 36.1% of patients were treated non-operatively whereas 63.9% of them had some type of surgical procedure: ORIF 25.7%, CRIF 22.3%, TSR 4.3% and PSR 11.6%. Non-surgical procedure were more frequent in men (39.5% vs, 34.9%; *p* < 0.01), and these patients were significantly older than patients treated with a surgical procedure [mean age 77.7 years (SD 7.2 years) vs 75.3 years (SD 6.3 years)], had higher mortality (2.3% vs 0.5%) and more IHC (3.2% vs 1.4%) and also had higher values of CCI. Overall women were more frequently treated with arthroplasty and men with non-operative treatment beside diabetes status (all *p* < 0.01).

The most common surgical procedure used in all groups was ORIF, followed by CRIF and arthroplasty. We observed a significant increase in the use of ORIF; among diabetic men, it increased from 9.5% in 2001 to 33.1% in 2013 (*p* < 0.01) and among those without the disease from 12.8% to 32.1% (*p* < 0.01). As is shown in Additional file 5: Table S1, for both diabetic and non-diabetic men, while ORIF increases in use during the study period, CRIF decreases (*p* < 0.05).

The IHM among men with T2DM decreased significantly over the study period, from 9.5% to 1.4% (*p* < 0.01). However, in those without T2DM IHM did not show a significant tendency (1.9% in 2001 to 1.3% in 2013). In non-diabetic men the median LOHS decreased significantly from 2001 to 2013.

ORIF was the most frequent procedure used for both groups of women. As observed for men, there was a significant increase in the use of ORIF, from 13.7% to 33.7% in non-diabetic women and from 15.2% to 31.6% in diabetic women, during the study period (*p* < 0.01). The use of CRIF decreased and the use of arthroscopy increased, in diabetic and non-diabetic women over time (*p* < 0.05). (Additional file 5: Table S1).

Diabetic women with PHF diagnosis reduceed their IHM significantly from 1.7% in 2001 to 1.0% in 2013 (*p* < 0.05). Over the 10-year study period, LOSH for women with and without T2DM decreased significantly (*p* < 0.05).

When we compared hospitalization outcomes between diabetic men and women we found almost double the crude IHM for men, compared to women, in the total study population. Among women those suffering T2DM had higher IHM than those without (1.4% vs. 0.8%; *p* < 0.05).

Table 3 summarizes the results of the multivariate logistic regression analysis of time trends and factors associated with IHM among men and women as a whole and with and without T2DM hospitalized for a PHF. Only factors with a clinical and statistical effect after the analysis are shown. LOHS was removed from the model, as it did not improve it.

**Table 3:** Multivariate analysis of the factors associated with in hospital mortality for proximal humerus fracture among men and women and stratified by type 2 diabetes in Spain. 2001–2013

		Men In-hospital mortality (OR)‡		Women In-hospital mortality (OR)‡		Whole study population In-hospital mortality (OR)	
Total		No T2DM	T2DM	No T2DM	T2DM	Men	Women
Age groups (years)	65–74	1	1	1	1	1	1
	75–79	1.45(0.86–2.43)	1.86(0.69–5.04)	2.07(1.24–3.46)	1.68(0.87–3.25)	1.56(0.98–2.47)	1.92(1.28–2.89)
	80–84	1.08(0.60–1.95)	1.67(0.57–4.88)	3.25(1.99–5.31)	2.48(1.30–4.73)	1.19(0.71–2.01)	3.05(2.06–4.52)
	85–89	3.73(2.22–6.26)	5.14(1.89–13.98)	5.40(3.31–8.82)	3.43(1.71–6.91)	4.25(2.67–6.78)	4.98(3.34–7.43)
	>90	2.80(1.31–5.97)	N/A	9.69(5.75–16.35)	7.72(3.72–16.02)	2.56(1.25–5.26)	9.90(6.44–15.21)
Charlson Comorbidity Index	0	1	1	1	1	1	1
	1	2.71(1.77–4.15)	2.52(1.0–6.35)	3.51(2.59–4.75)	3.95(2.43–6.44)	2.79(1.90–4.10)	3.70(2.86–4.78)
	≥ 2	6.29(3.85–10.28)	5.65(2.10–15.18)	6.7(4.47–10.10)	9.64(5.42–17.17)	6.66(4.30–10.32)	8.26(5.93–11.52)
In-hospital complications	No	1	1	1	1	1	1
	Yes	11.09(6.96–17.68)	10.10(4.21–24.25)	8.55(6.06–12.06)	6.20(3.68–10.45)	11.70(7.77–17.65)	8.21(6.15–10.96)
Arthroplasty	Yes	1	1	1	1	1	1
	No	9.36(2.88–30.42)	3.32(0.41–26.58)	3.16(1.81–5.54)	2.40(1.12–5.15)	7.14(2.57–19.83)	2.64(1.68–4.15)
ORIF	Yes	1	1	1	1	1	1
	No	5.72(2.73–11.96)	1.55(0.58–4.13)	4.51(2.58–7.89)	4.62(1.96–10.88)	3.67(2.07–6.52)	4.12(2.58–6.59)
CRIF	Yes	1	1	1	1	1	1
	No	2.46(1.45–4.19)	2.63(0.73–9.38)	2.08(1.42–3.04)	2.51(1.35–4.67)	2.44(1.5–3.98)	2.17(1.57–3.01)
Year of discharge		0.95(0.90–0.99)	0.82 (0.73–0.91)	0.93(0.90–0.97)	0.90(0.85–0.97)	0.92(0.88–0.97)	0.93(0.90–0.96)
T2DM status	No	NA	NA	NA	NA	1	1
	Yes	NA	NA	NA	NA	0.92(0.60–1.41)	1.67(1.29–2.15)

Arthroplasty: Total or partial humerus replacement, *ORIF* Open reduction of fracture with internal fixation, *CRIF* Closed reduction of fracture with internal fixation. Calculated using logistic regression models, Odds Ratio (OR). The logistic regression multivariate model was built using as dependent variables “death (yes/no)” and as independent variables year of discharge. Charlson comorbidity index. Complications. procedures and age

Among men and women with T2DM, IHM was significantly greater in older patients (OR 5.14; 95%CI, 1.89–13.98 for men in the 85–89 age group and OR 7.72; 95%CI, 3.72–16.02 in ≥90 women age group compared with the reference category 65–74 years).

IHM was significantly higher in diabetic men and women with more comorbidities (OR 5.65; 95%CI, 2.10–15.18 and OR 9.64; 95%CI, 5.42–17.17 for those with ≥2 comorbidities, respectively; *p* < 0.01) and in those with in-hospital complications (OR 10.10; 95%CI, 4.21–24.25 and OR 6.20; 95%CI, 3.68–10.45, for men and women, respectively: *p* < 0.01).

Arthroplasty, ORIF or CRIF were not associated to a higher mortality risk in diabetic women. In diabetic men the lower risk of mortality was not statistically significant for any of the procedures analyzed (Table 3).

Time trend analysis showed a significant decrease in mortality from 2001 to 2013 in diabetic men and women (OR 0.82; 95%CI, 0.73–0.91 and OR 0.90; 95%CI, 0.85–0.97; *p* < 0.05).

As can be seen in Table 3 the same variables were associated to IHM among men and women without T2DM.

When we performed logistic regression on our whole study population to assess the influence of T2DM on IHM and after adjusting for all covariates, being diabetic and suffering a PHF was associated to higher IHM in women (OR 1.67; 95%CI, 1.29–2.15; *p* < 0.05), but not in men (OR 0.92; 95%CI, 0.60–1.41).

## Discussion

We describe trends of incidence, in-hospital outcomes and surgical procedures for patients with and without T2DM with primary diagnosis of PHF admitted at Spanish hospitals from 2001 to 2013. Age-adjusted incidence rates increased steadily over the study period for men and women, independently of T2DM status, although we found a stable trend in the later years. Also we have found that patients with T2DM admitted to hospitals had lower IRR of PHF than non-T2DM patients hospitalized with PHF. However, in our investigation, women and men suffering T2DM showed a greater increase per year during the study period. We also describe a different pattern in treatment use over the time of study. ORIF shows an increasing use, while CRIF kept a decreasing tendency. However, total and partial arthroplasty use increased over time, for all the years and is significantly more frequently used in diabetic women than diabetic men. Regarding IHM, CCI ≥ 1 and 2, IHC and older age groups are associated with higher mortality. Having a surgical procedure was not associated to higher mortality.

Finally, presence of T2DM in women was associated to higher mortality, but not in men.

Our results confirm the increasing incidence of this type of fracture over time. This agrees with other authors [16, 27, 28]. Some of them predict a tremendous increase for 2030 (up to 50% comparing with 2008 incidence), partially because of increase in population age [28]. However, our results show a stable trend since 2007 in women and 2010 in men. This is consistent with results of some studies that found a stable incidence in PHF from 1999 to 2005, reporting, however, a 25% relative increase in the proportion of PHF treated surgically [5]. Kannur et al. described that among 80-year-old or older Finnish women the age-adjusted fracture rate of low-trauma fractures of the proximal humerus rose significantly from 1975 to 1995 and became stabilized from that year to 2015 [14].

The difference on sex incidence is described also by many other researches [4–7, 9–13]. Women have almost tripled the incidence of PHF as compared to men. This fact may be related with the greater prevalence of osteoporosis in women than men. Osteoporosis is related to more comminuted fracture pattern in which hemiarthroplasty is the preferred option [4, 16].

A possible explanation for the lower incidence of PHF among men suffering T2DM would be that diabetic men have protective factors for PHF in a higher frequency than non diabetic men. These factors include higher dietary calcium intake, higher use of bone pharmacologic interventions, practicing more physical exercise, less tobacco use and lower alcohol consumption among others [29, 30]. As we lack information on these variables further investigations are required to assess the effect of these uncontrolled confounders on the association found.

Regarding diabetes status, few studies describe use and outcomes of surgical procedures of humerus fracture repair in patients with T2DM. In one study with 325 patients with PHF, only 21% were treated surgically, and they found that only 10% had diabetes [9]. In our study we found that 18.3% of patients hospitalized suffered T2DM. Some studies describe higher risk of fracture in T2DM, with firm evidence for hip fractures [31, 32], however for PHF this is not so clear. It has been suggested that these results may be confounded by the higher fragility of diabetic persons, especially for women, and those patients with limited mobility and poor vision and therefore at greater risk of falling [32]. Diabetes is associated with an increased risk of falling more than once a year (OR 1.68 95%CI, 1.37–2.07) [32]. Moreover, patients with PHF have a slightly different risk factors pattern; for example obesity is described as a risk factor for PHF, but it is regarded as protective for hip fractures [33]. As we don’t have data on BMI we could not control this variable that could in part explain the lower incidence among diabetic men.

IHM showed a decreasing trend for men and women with T2DM, which was more significant than for non-diabetic patients. This is consistent with another study, which described a decreasing mortality rate, from 10.8% for men and 7.0% in women in 2008, to 8.5% for men and 6.4% for women in 2012 [11].

We have found two studies that analyze 30-day mortality among patients who have suffered PHF [34, 35]. In the US using the National Hospital Discharge Survey (NHDS) among all adult patients who were admitted to hospitals after fractures of the proximal humerus between 1990 and 2007 the in hospital mortality was 2.3% [34]. In Denmark among 5853 patients who underwent primary shoulder replacement, one-month mortality was 0.7% [35].

We found a higher IHM among diabetic men than diabetic women, which is compatible with other results that found a standardized mortality ratio of 1.6 (95%CI, 1.2–2.0) for women and 4.5 (95%CI, 3.3–6.1) for men [11]. However, another study found the same mortality rate in both sexes [36]. The higher mortality for men could be explained since diabetic women had significantly lower values of CCI and IHC than diabetic men.

In our study same patients with severe comorbidities, most frequently men, are considered for a non-operative treatment even if hospitalized. A possible explanation for this may be that, as upper arm fractures in men usually appear in cases of multiple injuries and they present higher mortality risk [11].

IHC and CCI values were higher among men and women with T2DM, than in patients without T2DM. This result agrees with others that state that diabetic patients have more comorbidity and suffer more complications during hospitalization [11, 37]. However, other authors do not agree [36, 38], as they have found that diabetes was not a predictor of adverse events among patients with PHF and reported a higher CCI as the risk factor for mortality and complications [38]. Therefore, it may be the presence of other comorbidities, some of which are very associated to T2DM (obesity), and not T2DM itself, the underlying reason for higher incidence of IHC among T2DM patients [33]. More studies are needed to clarify the effect of possible confounders on the in-hospital complications among diabetic patients.

Regarding the type of procedure used over the study period, we describe changes in the treatment for these fractures, in line with other studies. Most PHF are non-displaced or minimally displaced and they were treated non-operatively, but this pattern is clearly declining because of the technical and medical advantages of surgical treatment, which has increased steadily and has currently reached a plateau in the last few years [5, 16, 38].

Our results are consistent with other studies regarding the lower death risk seen in patients undergoing surgical treatment [36], but other studies do not agree [39]. Though a systematic review and meta-analysis did not find a significant difference between operative and non-operative treatment in health related quality of life [37], others did find a much higher risk of inpatient adverse events for ORIF and CRIF (OR 4.4; 95%CI, 4.3–4.6 and OR 2.7; 95%CI, 2.6–2.8, respectively) when compared to non-operative treatment. However patients treated surgically were less likely to be discharged to a long-term facility than patients treated non-operatively [39, 40].

Other studies have found a tendency to use hemiarthroplasty of the humerus head and total shoulder replacement to improve functional outcomes after treatment of comminuted (three or four segments) fractures, this seems to be also happening in Spain [6–9].

The advantage seen for surgical treatment with respect to lower mortality risk, was not so clear for diabetic men, which could be explained, as we stated previously, by the more complex fractures seen in men. Another explanation could be, as our results also have found, non-operative treatment, even of marginally displaced PHF in elderly people, is an important aspect of treatment, which seems to be considered increasingly in multimorbid patients. Our results showed that hospitalized patients that did not receive surgical treatment presented worse clinical characteristics, with higher CCI values, more complications, were older and they were more frequently men, resulting in significantly higher in-hospital mortality. Further studies are needed to clarify this point.

The strength of our investigation lies in its large sample size, its 13-year follow-up period and its standardized methodology, which has previously been used to investigate T2DM and its complications in Spain [22]. Nevertheless, our study is subject to a series of limitation. Our data source was the CMBD, an administrative database that contains discharge data for Spanish hospitalizations and uses information the physician has included in the discharge report. Coding practices, as well as errors in coding may differ between individual physicians and institutions. Thus our results are subject to several potential biases, including differences in capture of adverse outcomes across hospitals or even diabetes diagnosis along the study period.

Patients with T1DM were excluded because previous reports have shown that they have a different pattern of disease (disease duration, treatments, comorbidities) and epidemiological characteristics than T2DM patients [41]. Furthermore, different fracture risks and different mechanism of fracture between type 1 and T2DM patients have been reported [42, 43].

Other limitation is the lack of information on the characteristics of T2DM therapy used, the control reached, duration and other comorbid conditions that could affect the results. The database that we used contained no information about drugs treatments (i.e. drugs for osteoporosis) and risk factors such as obesity or smoke which may have affected our outcome variables.

As commented in the methods section patients who are treated at the emergency room or at out-patient clinic are not recorded in the SHNDD and therefore not included in our investigation. This may result in a selection bias, as those patients with less comorbidities and undergoing a non-operative treatment (immobilization) are more likely to be treated exclusively in the emergency room or as out patients and discharged without hospitalization. This can explain the very high proportion of PHF treated operatively (63.9%) in our study. However, it is also possible the existence of a bias by indication that would appear if the more advanced surgical treatment modalities were reserved for the more fit patients and similarly, patients with severe comorbidities were treated non-surgically.

Beside these limitations the quality and validity of our dataset has been assessed and shown to be useful for health research [44].

## Conclusions

The incidence of PHF seems to be increasing in Spain. The incidence is lower among men with than without T2DM. T2DM is associated to higher IHC in both sexes. The use of ORIF and arthroplasty is increasing overtime beside T2DM status. Women with T2DM have higher IHM than those without the disease.

## Additional files

Additional file 1: Figure S1. Joinpoint analysis of age-adjusted proximal humeral fractures hospitalizations in men without T2DM (Spain 2001–2013). Graph showing the Joinpoint analysis of age-adjusted proximal humeral fractures hospitalizations in men without T2DM (Spain 2001–2013). (TIFF 1794 kb)

Additional file 2: Figure S2. Joinpoint analysis of age-adjusted proximal humeral fractures hospitalizations in men with T2DM (Spain 2001–2013). Graph showing the Joinpoint analysis of age-adjusted proximal humeral fractures hospitalizations in men with T2DM (Spain 2001–2013). (TIFF 1897 kb)

Additional file 3: Figure S3. Joinpoint analysis of age-adjusted proximal humeral fractures hospitalizations in women without T2DM (Spain 2001–2013). Graph showing the Joinpoint analysis of age-adjusted proximal humeral fractures hospitalizations in women without T2DM (Spain 2001–2013). (TIFF 2051 kb)

Additional file 4: Figure S4. Joinpoint analysis of age-adjusted proximal humeral fractures hospitalizations in women with T2DM (Spain 2001–2013). Graph showing the Joinpoint analysis of age-adjusted proximal humeral fractures hospitalizations in women with T2DM (Spain 2001–2013). (TIFF 2373 kb)

Additional file 5: Table S1. Procedures and outcomes of hospital discharges due to proximal humerus fracture among men and women with and without type 2 diabetes in Spain, 2001–2013. Table showing the procedures and outcomes of hospital discharges due to proximal humerus fracture among men and women with and without type 2 diabetes in Spain, 2001–2013. (PDF 52 kb)

## Abbreviations

CCI: Charlson comorbidity index
CRIF: close reduction and internal fixation
HA: hemiarthroplasty
ICD-9-CM: International Classification of Diseases-Ninth Revision, Clinical Modification
IHC: in-hospital complications
IHM: in-hospital mortality
LOHS: length of stay
ORIF: open reduction and internal fixation
PSR: Partial shoulder replacement
SNHDD: Spanish National Hospital Discharge Database
TSR: total shoulder replacement
