# Supplementary figures and images for: Trends in incidence of proximal humerus fractures, surgical procedures and outcomes among elderly hospitalized patients with and without type 2 diabetes in Spain (2001–2013)
Source: BMC Musculoskelet Disord. 2017 Dec 11;18:522. doi: 10.1186/s12891-017-1892-7 (PMC5725839; doi:10.1186/s12891-017-1892-7)

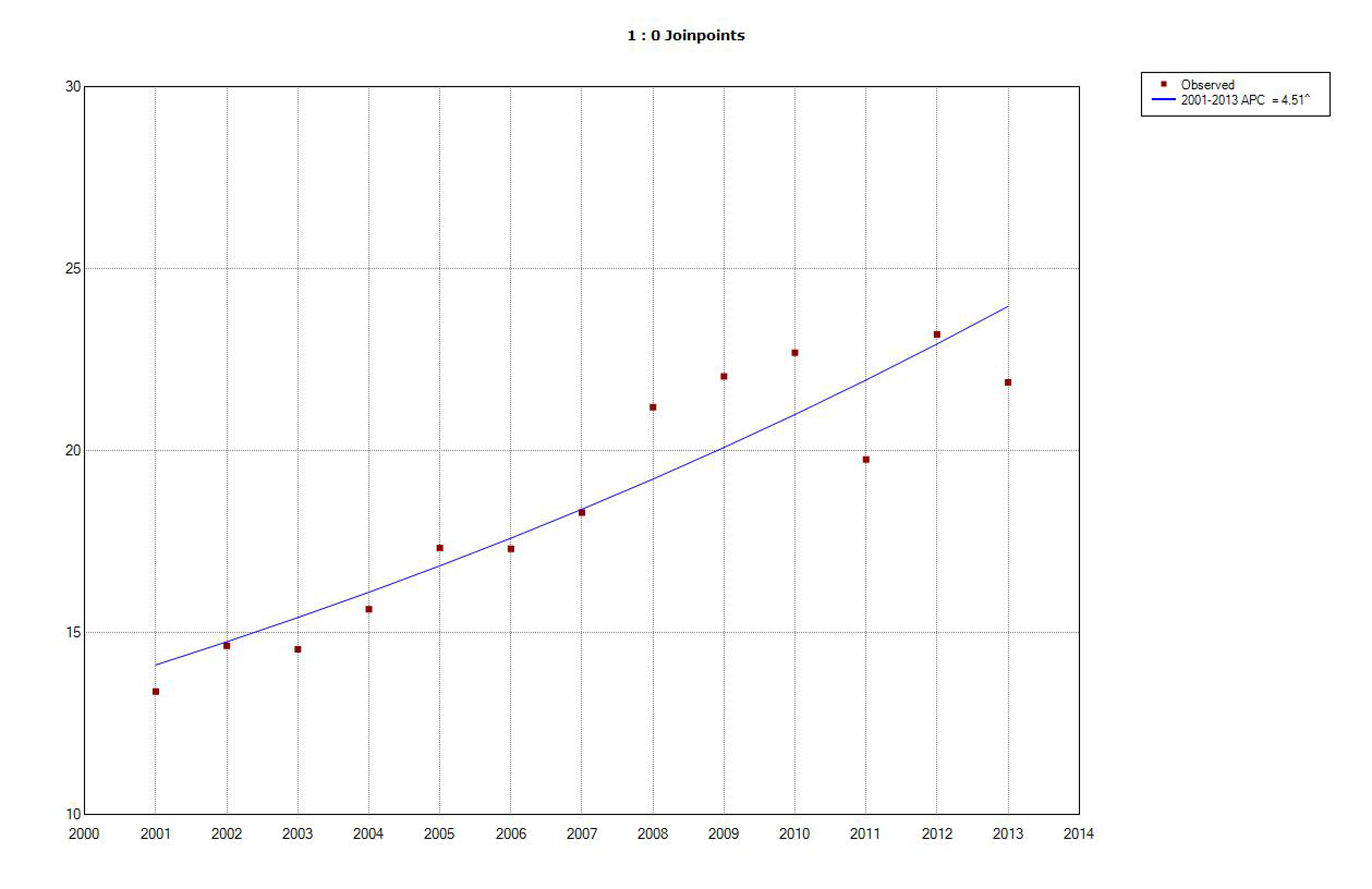

Supplement: Supplementary file 1 — Joinpoint analysis of age-adjusted proximal humeral fractures hospitalizations in men without T2DM (Spain 2001–2013). Graph showing the Joinpoint analysis of age-adjusted proximal humeral fractures hospitalizations in men without T2DM (Spain 2001–2013). (TIFF 1794 kb) [file 12891_2017_1892_MOESM1_ESM.tif]

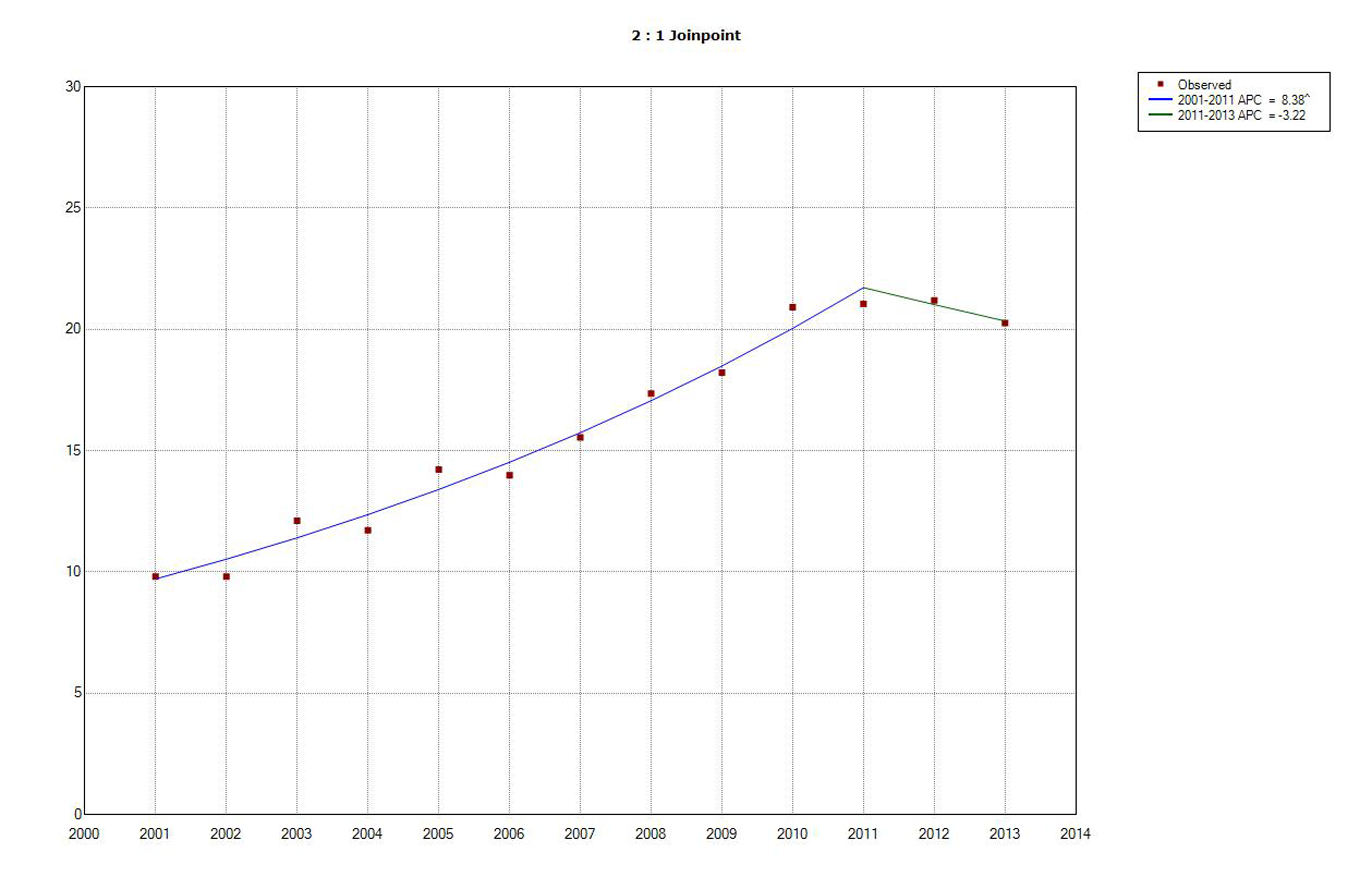

Supplement: Supplementary file 2 — Joinpoint analysis of age-adjusted proximal humeral fractures hospitalizations in men with T2DM (Spain 2001–2013). Graph showing the Joinpoint analysis of age-adjusted proximal humeral fractures hospitalizations in men with T2DM (Spain 2001–2013). (TIFF 1897 kb) [file 12891_2017_1892_MOESM2_ESM.tif]

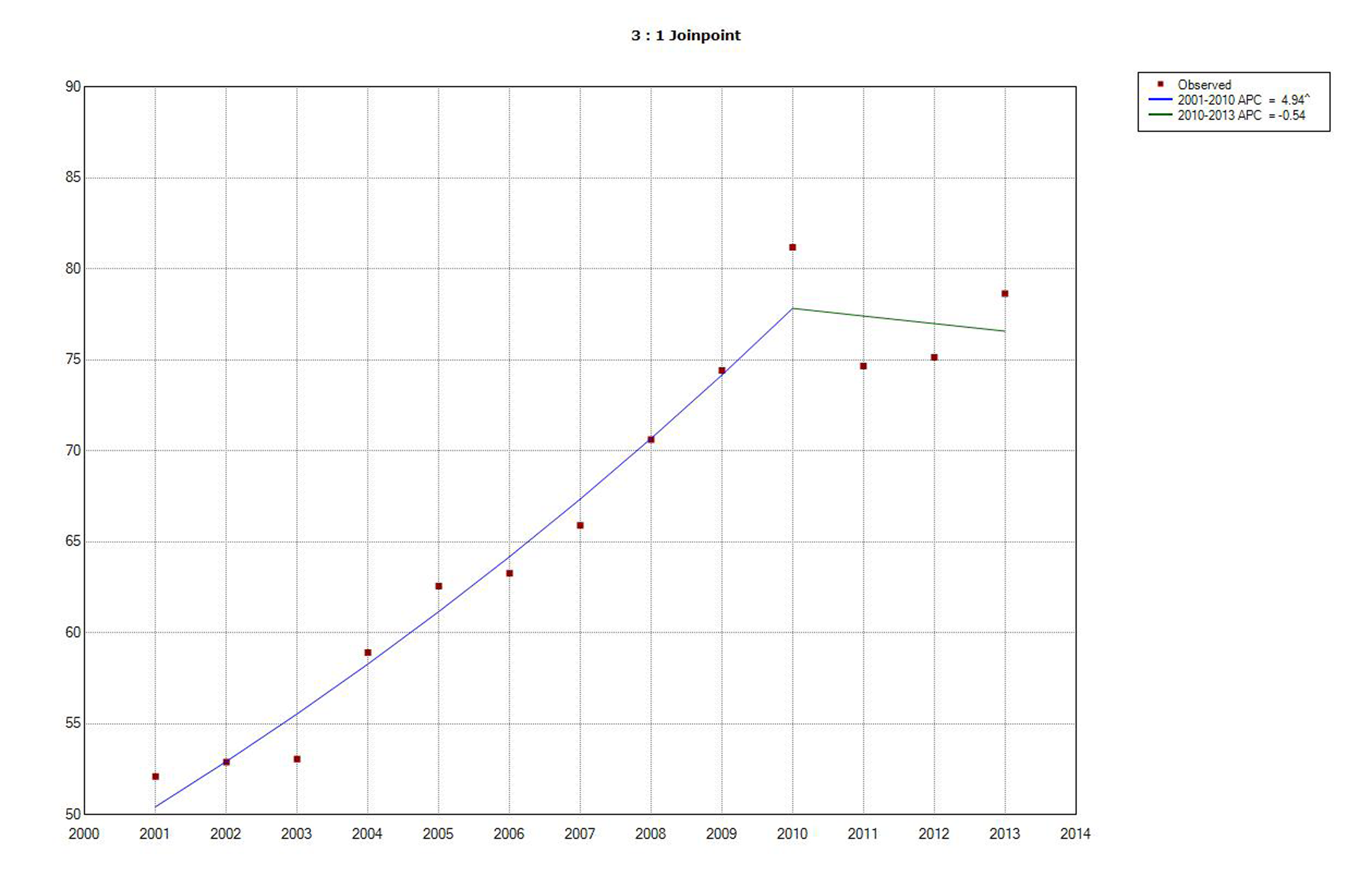

Supplement: Supplementary file 3 — Joinpoint analysis of age-adjusted proximal humeral fractures hospitalizations in women without T2DM (Spain 2001–2013). Graph showing the Joinpoint analysis of age-adjusted proximal humeral fractures hospitalizations in women without T2DM (Spain 2001–2013). (TIFF 2051 kb) [file 12891_2017_1892_MOESM3_ESM.tif]

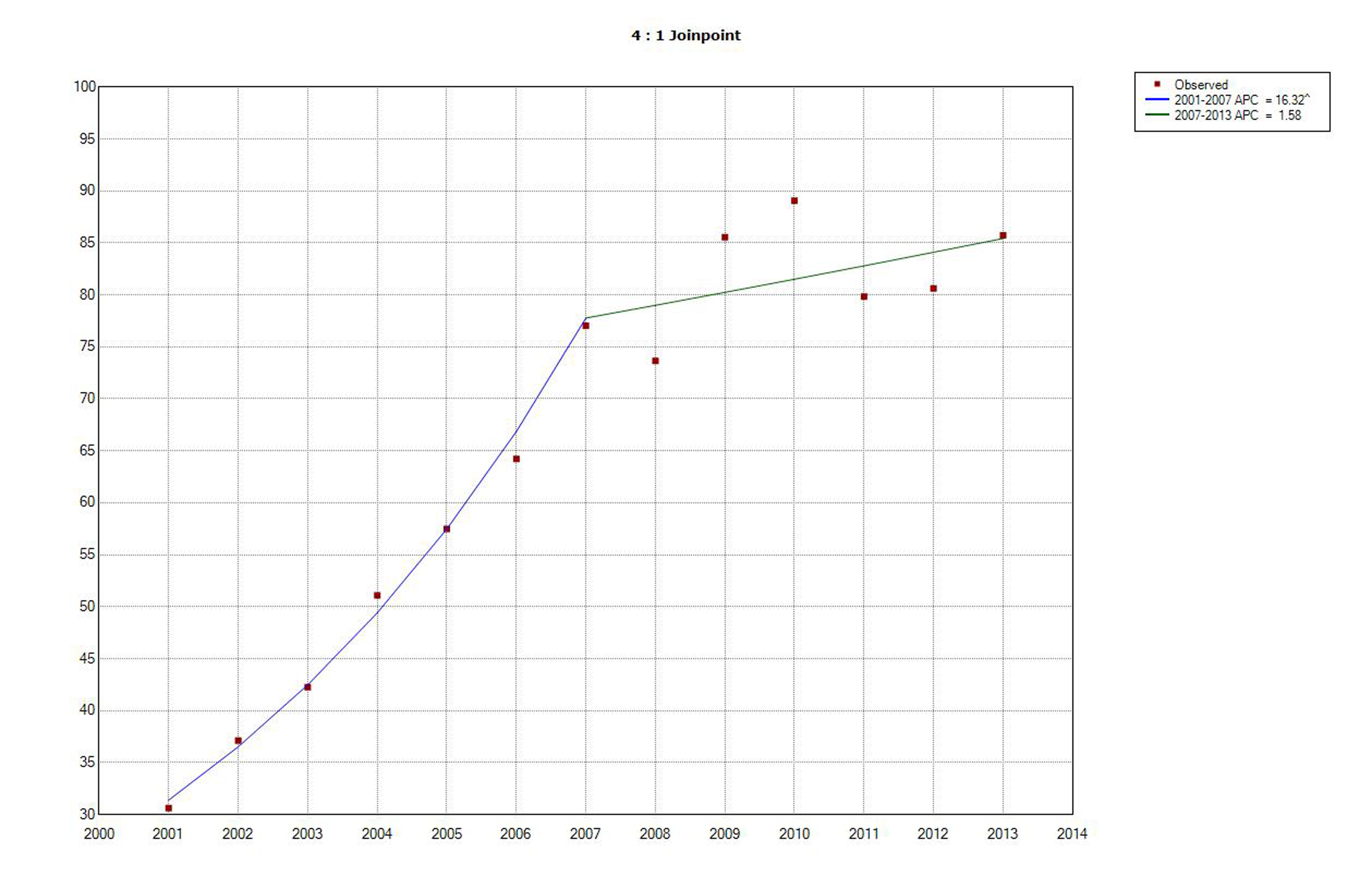

Supplement: Supplementary file 4 — Joinpoint analysis of age-adjusted proximal humeral fractures hospitalizations in women with T2DM (Spain 2001–2013). Graph showing the Joinpoint analysis of age-adjusted proximal humeral fractures hospitalizations in women with T2DM (Spain 2001–2013). (TIFF 2373 kb) [file 12891_2017_1892_MOESM4_ESM.tif]
