# Supplementary material for: Trends in incidence of proximal humerus fractures, surgical procedures and outcomes among elderly hospitalized patients with and without type 2 diabetes in Spain (2001–2013)
Source: BMC Musculoskelet Disord. 2017 Dec 11;18:522. doi: 10.1186/s12891-017-1892-7 (PMC5725839; doi:10.1186/s12891-017-1892-7)
Supplement: Supplementary file 5 — Procedures and outcomes of hospital discharges due to proximal humerus fracture among men and women with and without type 2 diabetes in Spain, 2001–2013. Table showing the procedures and outcomes of hospital discharges due to proximal humerus fracture among men and women with and without type 2 diabetes in Spain, 2001–2013. (PDF 52 kb) [file 12891_2017_1892_MOESM5_ESM.pdf]

Supplementary table 1. Procedures and outcomes of hospital discharges due to proximal humerus fracture among men and women with and without type 2 diabetes in Spain, 2001-2013

	2001	2002	2003	2004	2005	2006	2007	2008	2009	2010	2011	2012	2013	Total
**MEN WITHOUT T2DM**														
Arthroplasty, n (%)[ba]	27(8.4)	47(13.4)	38(10.6)	47(12.2)	57(13.2)	58(13.3)	58(12.6)	66(12.2)	80(13.9)	99(16.9)	72(13.6)	105(16.9)	115(19.3)	869(14.0)
ORIF, n (%)[a]	41(12.8)	50(14.3)	52(14.6)	73(19.0)	109(25.3)	90(20.6)	113(24.5)	161(29.7)	152(26.5)	181(30.6)	189(35.7)	195(31.4)	191(32.1)	1597(25.8)
CRIF, n (%)[a]	76(23.8)	86(24.6)	98(27.5)	98(25.5)	97(22.5)	111(25.5)	104(22.6)	105(19.3)	118(20.6)	102(17.3)	105(19.8)	105(16.9)	94(15.8)	1299(21.0)
NON-OPERATIVE, n (%)[a]	177(55,3)	169(48.3)	170(47.6)	169(44.0)	170(39.4)	183(42.0)	189(41.0)	217(40.0)	227(39.5)	211(35.7)	168(31.7)	222(35.7)	199(33.4)	2471(39.2)
IHM, n (%)	6(1.9)	9(2.6)	8(2.2)	7(1.8)	14(3.2)	16(3.7)	9(2.0)	13(2.4)	13(2.3)	12(2.0)	8(1.5)	16(2.6)	8(1.3)	139(2.2)
LOHS, median (IRQ)[a]	5(2-11)	5(3-10)	6(2-11)	5.5(3-11)	6(3-11)	6(3-10.7)	6(3-10)	6(3-11)	5(2-10)	5(3-9)	5(3-9)	4(2-8)	4(2-9)	5(2-10)
**MEN WITH T2DM**														
Arthroplasty, n (%)	6(14.3)	5(11.9)	4(6.6)	7(11.9)	8(10.4)	8(9.9)	10(11.1)	18(17.1)	13(11.3)	15(11.4)	13(8.8)	17(11.5)	19(12.8)	143(11.5)
ORIF, n (%)[a]	4(9.5)	5(11.9)	9(14.8)	10(16.9)	23(29.9)	15(18.5)	20(22.2)	29(27.6)	35(30.4)	46(34.8)	42(28.6)	48(32.4)	49(33.1)	335(26.9)
CRIF. n (%)	7(16.7)	14(33.3)	17(27.9)	16(27.1)	15(19.5)	17(21.0)	20(22.2)	22(21.0)	24(20.9)	23(17.4)	38(25.9)	28(18.9)	26(17.6)	267(21.4)
NON-OPERATIVE, n (%)[a]	26(61.9)	18(42.9)	31(50.8)	28(47.5)	32(41.6)	41(50.6)	41(45.6)	37(35.2)	44(38.3)	48(36.4)	55(37.4)	56(37.8)	54(36.5)	511(40.2)
IHM, n (%)[a]	4(9.5)	1(2.4)	1(1.6)	1(1.7)	4(5.2)	3(3.7)	1(1.9)	2(1.9)	5(4.3)	3(2.3)	2(1.4)	2(1.4)	2(1.4)	31(2.5)
LOHS, median (IRQ)	7.5(3-12.5)	7(3-12)	6(2-10)	5(2-14)	7(3-14)	6(4-11)	7(3-11.2)	7(3-13)	6(4-11)	6(3-11)	6(3-10)	4(2-9)	4(2-9)	6(3-11)
**WOMEN WITHOUT T2DM**														
Arthroplasty, n (%)[a, c]	204(12.7)	221(13.4)	232(13.4)	277(14.4)	282(13.4)	316(14.5)	368(16.2)	415(16.9)	448(17.1)	484(17.0)	520(19.4)	535(19.8)	581(20.2)	4883(16.1)
ORIF, n (%)[a, c]	220(13.7)	262(16.1)	300(17.3)	354(18.4)	434(20.7)	526(24.1)	552(24.3)	715(29.1)	770(29.4)	900(31.5)	838(31.2)	915(33.9)	970(33.7)	7756(26.2)
CRIF, n (%)[a]	457(28.5)	440(27.0)	500(28.9)	502(26.1)	538(25.6)	567(26.0)	525(23.1)	534(21.7)	547(20.9)	580(20.3)	561(20.9)	454(16.8)	514(17.9)	6719(22.1)
NON-OPERATIVE, n (%)[a]	730(45.5)	711(43.6)	711(41.1)	803(41.8)	851(40.6)	786(36.1)	835(36.8)	812(33.0)	874(33.4)	906(31.7)	785(29.2)	813(30.1)	830(28.8)	10447(35.0)
IHM, n (%)[a, c c]	10(0.6)	14(0.9)	20(1.2)	16(0.8)	24(1.1)	20(0.9)	19(0.8)	22(0.9)	15(0.6)	21(0.7)	16(0.6)	16(0.6)	19(0.7)	232(0.8)
LOHS, median (IRQ) [a]	5(2-9)	5(3-10)	5(3-9)	5(3-9)	5(3-10)	6(3-10)	6(3-10)	6(3-10)	5(3-9)	5(3-9)	5(2-8)	5(2-8)	4(2-8)	5(3-9)
**WOMEN WITH T2DM**														
Arthroplasty, n (%)[a]	37(16.0)	42(15.0)	52(16.7)	58(15.4)	70(16.7)	65(14.1)	93(16.8)	93(16.6)	124(18.0)	137(19.2)	118(16.6)	145(20.2)	184(23.6)	1218(17.7)
ORIF, n (%)[a]	35(15.2)	36(12.9)	50(16.1)	62(16.5)	73(17.5)	120(26.0)	123(22.2)	131(23.4)	170(24.7)	213(29.8)	214(30.1)	218(30.3)	246(31.6)	1691(24.1)
CRIF, n (%)[a]	65(28.1)	80(28.6)	82(26.4)	107(28.5)	109(26.1)	119(25.8)	137(24.8)	133(23.8)	145(21.1)	143(20.0)	164(23.0)	145(20.2)	117(15.0)	1546(22.6)
NON-OPERATIVE, n (%)[a]	99(42.9)	126(45.0)	130(41.8)	150(39.9)	170(40.7)	159(34.5)	205(37.1)	205(36.6)	249(36.2)	225(31.5)	222(31.2)	215(29.9)	241(30.9)	2396(34.6)
IHM, n (%)[a]	4(1.7)	4(1.4)	5(1.6)	12(3.2)	10(2.4)	7(1.5)	7(1.3)	6(1.1)	7(1.0)	7(1.0)	10(1.4)	9(1.3)	8(1.0)	96(1.4)
LOHS, median (IRQ) [a]	6(3-12)	7(4-12.7)	6(3-12)	6(3-10)	7(4-10.2)	7(4-11)	7(3-11)	6(4-11)	6(3-10)	5(3-9)	5(3-8)	5(2-9)	5(2-9)	6(3-10)

Arthroplasty Total or partial humerus replacement; ORIF: Open reduction of fracture with internal fixation ; CRIF: Close reduction of fracture with internal fixation; IHM: In-hospital mortality;;LOHS: Length of hospital stay.[a] $P<0.05$ for time trend. [b] . $P<0.05$ when comparing total values of study variables between men with and without T2DM. [c] . $P<0.05$ when comparing total values of study variables between women with and without T2DM. Stadistical tsts are the same than in Table 1 and 2.
